# Outpatient Versus Hospital-Based Total Knee Arthroplasty: A Survey-Based Comparison of Complications, Infection, and Patient Satisfaction in a Contemporary U.S. Cohort

**DOI:** 10.7759/cureus.107598

**Published:** 2026-04-23

**Authors:** Braden R Woo, Muhammad Yamaan Khan, Krishn B Patel, Nadiya A Persaud, Linda Brecher

**Affiliations:** 1 Research, Orlando College of Osteopathic Medicine, Winter Garden, USA; 2 College of Public Health, University of South Florida, Tampa, USA

**Keywords:** outpatient surgery, patient satisfaction, postoperative complications, robotic-assisted surgery, total knee arthroplasty

## Abstract

Introduction

The use of outpatient pathways for total knee arthroplasty (TKA) has expanded with advances in perioperative optimization and risk stratification; however, concerns remain regarding safety and patient outcomes. This study aimed to compare postoperative complications, infection rates, and patient satisfaction between an outpatient surgery center and hospital-based TKA, while assessing the relative contribution of patient-level factors to outcomes.

Methodology

A cross-sectional survey of 384 adults in the United States who had undergone primary TKA was conducted. Individuals with additional joint arthroplasties were excluded to isolate the effect of the surgical setting, resulting in 305 hospital TKAs and 79 outpatient TKAs. The primary exposure was a surgical setting. Primary outcomes included any self-reported postoperative complication, infection, and willingness to undergo TKA again. Outcomes were compared using chi-square and t-tests. Multivariable logistic regression was performed, adjusting for age, sex, body mass index (BMI), diabetes, surgical technique, and physical therapy adherence.

Results

Outpatient TKA patients were younger (47.6 vs. 55.4 years, *P* < 0.00001) and more frequently male (55.7% vs. 40.3%, *P* = 0.0199). These differences reflect real-world patient selection patterns for outpatient arthroplasty and indicate baseline imbalance between cohorts. Diabetes prevalence did not differ significantly between groups (17.4% vs. 13.9%, *P* = 0.57). Rates of complications (34.2% vs. 31.8%, *P* = 0.79), infection (5.1% vs. 8.5%, *P* = 0.43), and willingness to undergo TKA again (82.3% vs. 77.0%, *P* = 0.40) were similar between outpatient and hospital settings. Among older patients (≥56 years), outpatient TKA was associated with a higher proportion of patients willing to undergo the procedure again (91.3% vs. 75.4%).

In adjusted analyses, surgical setting was not associated with complications (odds ratio (OR) 0.83, 95% confidence interval (CI) 0.47-1.47, *P* = 0.52), infection (OR 0.41, 95% CI 0.13-1.27, *P* = 0.12), or satisfaction (OR 1.21, 95% CI 0.61-2.43, *P* = 0.58). Higher BMI (OR 1.07, 95% CI 1.03-1.11, *P* = 0.0012) and male sex (OR 0.26 for female vs. male, 95% CI 0.11-0.61, *P* = 0.0021) were associated with increased infection risk. Robotic-assisted TKA (OR 3.29, 95% CI 1.43-7.57, *P* = 0.0052) and male sex (OR 0.47 for female vs. male, 95% CI 0.27-0.82, *P* = 0.0078) were associated with higher satisfaction. Diabetes was associated with increased complications (OR 1.95, 95% CI 1.06-3.57, *P* = 0.030) and lower satisfaction (OR 0.47, 95% CI 0.24-0.92, *P* = 0.027), but not infection.

Conclusions

Outpatient TKA was associated with similar observed rates of complications, infection, and patient satisfaction compared with hospital-based TKA in this cohort; however, these findings should be interpreted with caution, given baseline differences between groups and the potential for residual confounding. Patient-level factors, including BMI, sex, diabetes, and use of robotic assistance, appear to have a greater impact on outcomes than surgical setting. These findings support outpatient TKA as a safe and effective approach and emphasize the importance of individualized risk stratification and perioperative optimization.

## Introduction

The demand for total knee arthroplasty (TKA) continues to grow as the population ages, and the prevalence of knee osteoarthritis increases [1]. Primary TKA volume in the United States is projected to reach approximately 935,000 procedures annually by 2030, underscoring the importance of developing efficient and safe care pathways for this high-volume procedure [2]. Advances in perioperative optimization, multimodal analgesia, blood conservation strategies, and enhanced recovery protocols have made shorter hospital stays and even same-day discharge (SDD) increasingly feasible for appropriately selected, lower-risk patients [3]. Multiple risk stratification tools, including the Outpatient Arthroplasty Risk Assessment (OARA) score, have been developed and validated to support the safe selection of patients for outpatient joint arthroplasty [4-5].

Historically, joint replacement procedures required prolonged hospitalization, with the earliest total hip replacements necessitating admissions of up to three months. As recently as two decades ago, patients typically remained in the hospital for approximately five days following hip, knee, or shoulder replacement, with length of stay decreasing to two to three days within the subsequent decade [6]. Over this same period, the number and utilization of ambulatory surgery centers (ASCs) for TKA have increased dramatically, facilitating a shift from inpatient care to SDD for many patients and largely replacing the traditional three- to four-day hospital stay [7].

Outpatient TKA has emerged as an attractive model from both health system and patient perspectives. Analyses of large national databases, including the American College of Surgeons National Surgical Quality Improvement Program (ACS NSQIP), along with economic evaluations, have demonstrated that outpatient or short-stay TKA is associated with reduced healthcare costs and comparable, and in some cases improved, patient satisfaction when appropriate patient selection criteria are applied, including lower comorbidity burden, favorable anesthetic risk profile (e.g., lower American Society of Anesthesiologists class), functional independence, and adequate social support, often guided by validated risk stratification tools such as the OARA score [5,8-9].

However, the literature evaluating the safety and complication profile of outpatient TKA remains mixed. A 2020 meta-analysis reported higher complication rates in outpatient TKA compared with inpatient procedures, raising concerns regarding the generalizability of outpatient protocols and the adequacy of current patient selection criteria [10]. In contrast, more recent studies employing fast-track protocols and propensity-matched designs have reported no increase in complication rates among outpatient cohorts, suggesting that, under optimized conditions, outpatient TKA is not inherently associated with greater risk compared with inpatient care [11]. Comparable, and in some cases improved, outcomes have been reported among carefully selected patients undergoing outpatient total hip and knee arthroplasty [12]. Importantly, many existing studies rely on administrative datasets, institutional cohorts, or registry-based outcomes, which may not fully capture patient-reported satisfaction or the complexity of post-discharge complications as experienced in real-world settings [13-15]. Furthermore, variability in the measurement and implementation of patient-reported outcomes, along with heterogeneity in patient selection criteria, perioperative protocols, and institutional practice patterns, likely contributes to the inconsistencies observed across reported outcomes [16].

To further contribute to this evolving body of evidence, a survey was conducted among patients in the United States who underwent primary TKA, with results analyzed to directly compare outcomes between hospital-based and outpatient surgery center settings. The primary aim of this study was to evaluate the association between surgical setting (outpatient vs. hospital-based) and patient-reported postoperative outcomes, including complications, infection, and satisfaction, within a contemporary U.S. cohort. This study was not designed to establish equivalence or non-inferiority between settings, but rather to characterize observed outcome patterns in a real-world population. A secondary objective was to assess the relative contribution of patient-level characteristics, including body mass index (BMI), sex, diabetes status, and use of robotic-assisted techniques, to postoperative outcomes in comparison with the surgical setting. The use of patient-reported outcomes was intended to capture patient-centered experiences that may not be fully reflected in administrative datasets, while acknowledging the inherent limitations of self-reported data.

## Materials and methods

A cross-sectional observational study was conducted using a survey distributed to adults in the United States (Appendix). Respondents provided demographic information, surgical details including operative setting and technique, and self-reported postoperative outcomes and experiences. A survey-based approach was selected to capture patient-reported outcomes across a geographically diverse population, offering insight into real-world postoperative perceptions that may not be fully represented in registry or institutional datasets. However, this approach is subject to recall bias and lacks standardization of perioperative protocols across respondents. The study was reviewed by the Orlando College of Osteopathic Medicine Institutional Review Board and deemed minimal risk in accordance with NIH guidelines (OCOM-E-2025-0004).

The survey was distributed using a random device engagement methodology, a digital sampling approach in which survey prompts are delivered to a randomized pool of active devices across a broad geographic region. This method is intended to enhance population diversity and reduce selection bias associated with single-institution or convenience sampling, although it does not allow for direct verification of clinical data or standardized follow-up.

A screener question was used to identify individuals who reported having undergone primary TKA and who specified the location of surgery as either a hospital or an outpatient surgery center. To isolate the effect of surgical setting and minimize confounding, respondents who reported additional joint arthroplasties (including total hip, ankle, shoulder, reverse shoulder, or wrist arthroplasty) were excluded. The final analytic sample consisted of 384 patients, including 305 who underwent TKA in a hospital setting and 79 in an outpatient surgery center.

The primary exposure variable was surgical setting, categorized as hospital-based versus outpatient surgery center. Outpatient procedures were defined as those performed in freestanding or affiliated ASCs with SDD or short-stay discharge. For regression analyses, the surgical setting was treated as a binary variable.

Three primary outcomes were evaluated: (1) any postoperative complication, (2) postoperative infection, and (3) willingness to undergo TKA again as a measure of global patient satisfaction. Any complication was defined as the presence of one or more self-reported postoperative complications, excluding responses indicating no complications. Infection was analyzed as a specific complication. Complications and infection were defined based on respondent-selected options within the survey instrument and were not independently verified or adjudicated using standardized clinical criteria. Willingness to undergo the procedure again was derived from a binary survey response. Secondary patient-reported outcomes included preoperative and postoperative pain, joint stiffness, functional improvement (including ability to exercise), adherence to postoperative physical therapy, and satisfaction with physical therapy. These variables were summarized descriptively and evaluated in unadjusted analyses.

All outcomes were based on patient self-report and were not derived from validated functional outcome instruments such as the Western Ontario and McMaster Universities Osteoarthritis Index (WOMAC) or the Knee Injury and Osteoarthritis Outcome Score (KOOS). Accordingly, satisfaction was operationalized as a global, patient-centered measure using a single binary question rather than a multidimensional validated scale.

Potential confounders included age, sex, BMI, diabetes status, and surgical technique (categorized as robotic-assisted vs. conventional). BMI was calculated from self-reported height and weight using the standard formula. Responses indicating uncertainty regarding surgical technique were treated as missing in regression analyses. Physical therapy adherence was included as a continuous variable.

Descriptive statistics were reported as means with standard deviations for continuous variables and counts with percentages for categorical variables. Unadjusted comparisons between hospital and outpatient groups were performed using Student’s t-tests for continuous variables and chi-square tests for categorical variables. Subgroup analyses examined willingness to undergo TKA again across strata of sex and age, dichotomized at the median age of 56 years.

Multivariable logistic regression models were constructed to evaluate the association between surgical setting and each primary outcome, adjusting for age, sex, BMI, diabetes status, surgical technique, and physical therapy adherence. Adjusted odds ratios (ORs) with 95% confidence intervals (CIs) and corresponding *P*-values were reported. Statistical significance was defined using a two-sided alpha level of 0.05, and no adjustment was made for multiple comparisons. All statistical analyses were performed using RStudio (version 2024.12.1+563, Posit, Boston, MA) with R version 4.4.1 (R Foundation for Statistical Computing, Vienna, Austria).

## Results

Cohort characteristics

The final analytic sample included 384 (100%) respondents with isolated TKA, of whom 305 (79.4%) underwent surgery in a hospital setting and 79 (20.6%) in an outpatient surgery center (Table 1). Outpatient TKA patients were significantly younger than hospital TKA patients (mean age 47.6 ± 13.1 vs. 55.4 ± 14.9 years, P < 0.00001) and more likely to be male (44 (55.7%) vs. 123 (40.3%), *P* = 0.0199). Mean BMI was in the obese range for both groups (32.7 ± 9.0 kg/m² for hospital vs. 30.9 ± 8.9 kg/m² for outpatient). Diabetes prevalence was similar between groups 11 (13.9%) vs. 53 (17.4%), *P* = 0.57. Surgical technique included both conventional and robotic-assisted approaches, with robotic-assisted TKA performed in 53/304 (17.4%) hospital cases and 17/79 (21.5%) outpatient cases (Table 1).

**Table 1:** Baseline characteristics by surgical setting. SD, standard deviation; BMI, body mass index; TKA, total knee arthroplasty

Characteristics	Hospital (*n* = 305)	Outpatient center (*n* = 79)
Age at procedure, mean ± SD (years)	55.4 ± 14.9	47.6 ± 13.1
Female, *n* (%)	182 (59.7%)	35 (44.3%)
Male, *n* (%)	123 (40.3%)	44 (55.7%)
BMI (mean ± SD) (kg/m²)	32.7 ± 9.0	30.9 ± 8.9
Diabetes, *n* (%)	53 (17.4%)	11 (13.9%)
Robotic-assisted TKA, *n* (%)	53/304 (17.4%)	17/79 (21.5%)

Perioperative characteristics

**Figure 1:**
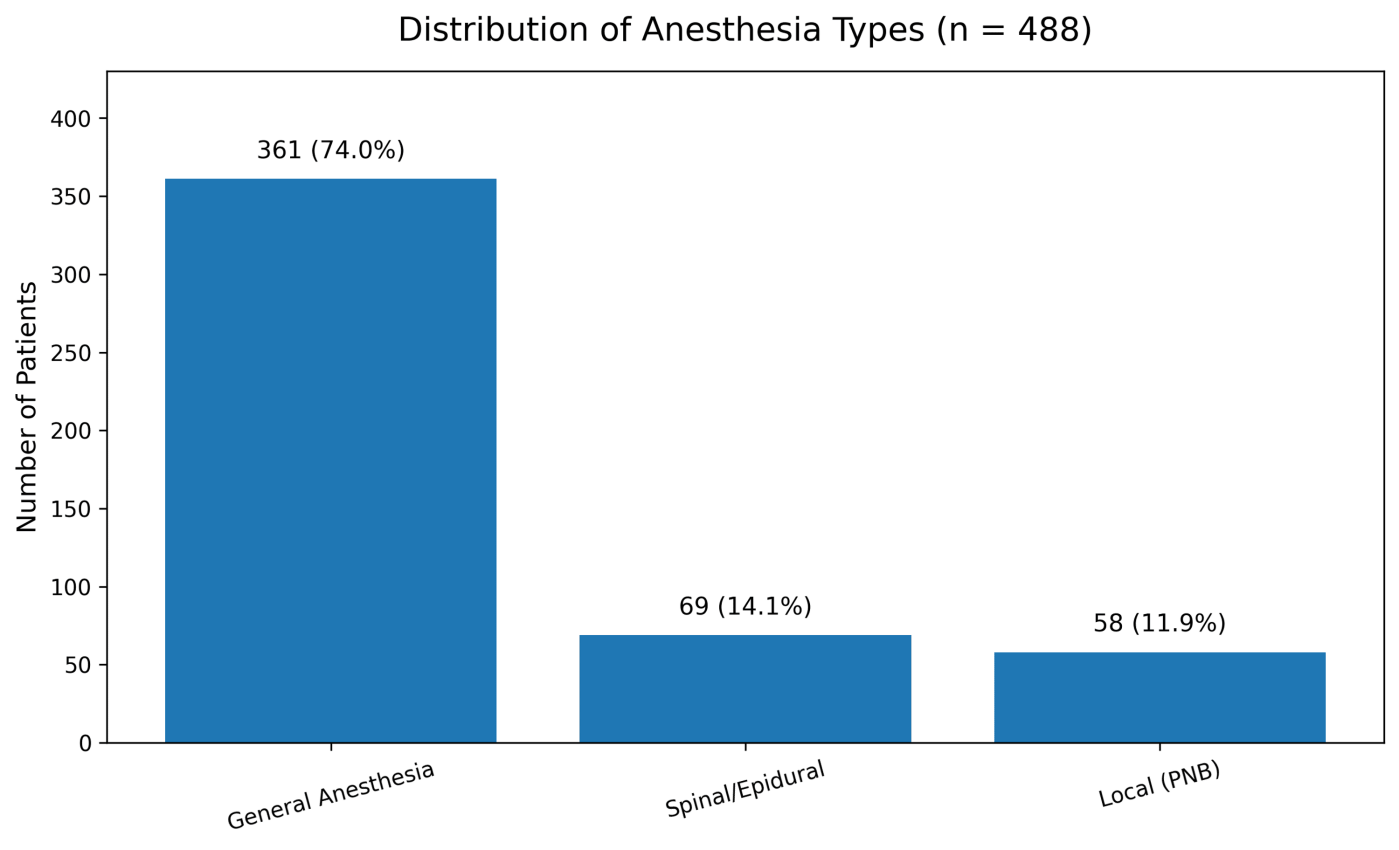
Distribution of anesthesia modalities for TKA, demonstrating the predominance of general anesthesia, with smaller proportions undergoing spinal/epidural or peripheral nerve block techniques. TKA, total knee arthroplasty

Anesthesia type varied across respondents, with general anesthesia being the most commonly utilized modality, followed by spinal or epidural anesthesia and peripheral nerve block or local anesthesia (Figure 1).

Preoperative and postoperative patient-reported outcomes

**Figure 2:**
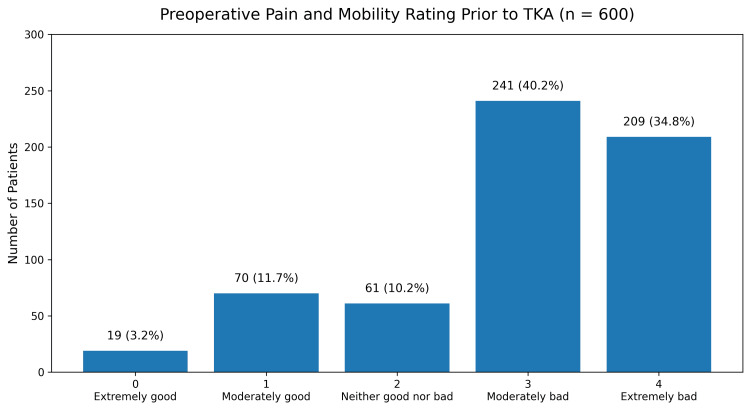
Patient-reported pain and mobility outcomes following TKA, demonstrating significant improvement compared with preoperative status. TKA, total knee arthroplasty

Preoperatively, patients reported substantial impairment in joint function, with the majority describing pain, mobility, and stiffness as moderate to severe. Following TKA, marked improvements were observed in patient-reported outcomes. Most respondents reported postoperative pain and mobility as moderately good or extremely good, with only a minority reporting persistent moderate or severe impairment (Figure 2). Similarly, postoperative joint stiffness improved substantially, with the majority reporting favorable outcomes.

Functional improvement

Most patients reported improved ability to exercise following TKA. In the isolated TKA cohort, 288 respondents reported improvement, while 49 reported no change and 47 reported worsening. Data regarding preoperative osteoarthritis severity, such as radiographic grading (e.g., Kellgren-Lawrence classification), were not collected, and therefore, the correlation between arthritis grade and functional improvement could not be assessed.

Rehabilitation and physical therapy

Rehabilitation settings varied among respondents. Outpatient rehabilitation centers (151, 25.17%) and combination at-home and outpatient rehabilitation (156, 26.00%) were the most commonly reported approaches, followed by at-home rehabilitation alone (147, 24.50%). Smaller proportions reported inpatient rehabilitation (64, 10.67%), combination at-home and inpatient rehabilitation (50, 8.33%), or combination inpatient and outpatient rehabilitation (24, 4.00%). Adherence to physical therapy was generally high, with 174 respondents (29.00%) reporting the highest adherence level and 266 (44.33%) reporting good adherence. Satisfaction with physical therapy was also favorable, with 214 respondents (35.67%) reporting moderate satisfaction and 193 (32.17%) reporting high satisfaction.

Postoperative complications

**Figure 3:**
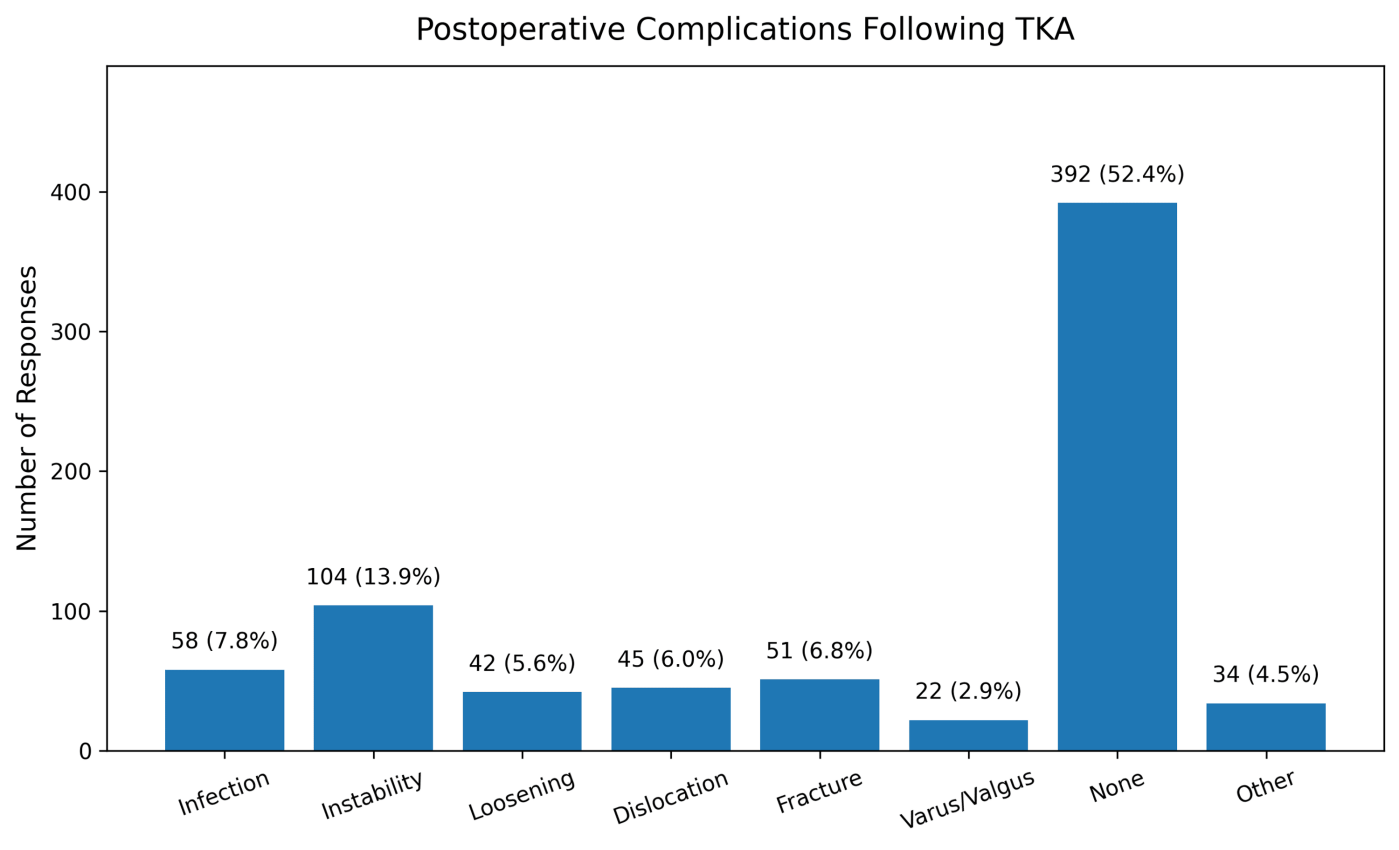
Distribution of self-reported postoperative complications following TKA, with instability and infection among the most frequently reported events. Responses reflect a select-all-that-apply question. TKA, total knee arthroplasty

Overall, 392 (52.4%) respondents reported no postoperative complications. Among those who experienced complications, the most commonly reported were instability, infection, fracture, dislocation, and implant loosening (Figure 3). Data regarding perioperative and postoperative antibiotic protocols were not collected, and therefore, their potential impact on infection outcomes could not be evaluated.

Unadjusted outcomes by surgical setting

Overall complication rates were similar between settings, occurring in 97 (31.8%) of 305 hospital TKA patients and 27 (34.2%) of 79 outpatient TKA patients (χ²(1) = 0.07, *P* = 0.79). Infection rates were 26/305 (8.5%) for hospital TKA patients and 4/79 (5.1%) for outpatient TKA patients (χ²(1) = 0.62, *P* = 0.43) (Table 2). Willingness to undergo TKA again was high in both groups, reported by 235/305 (77.0%) hospital TKA patients and 65/79 (82.3%) outpatient TKA patients (χ²(1) = 0.72, P = 0.40). None of these unadjusted comparisons reached statistical significance.

**Table 2:** Unadjusted primary outcomes following TKA by surgical setting. Values are presented as counts and percentages, with corresponding p-values for comparisons between hospital and outpatient surgery center groups. TKA, total knee arthroplasty

Outcome	Hospital (*n* = 305)	Outpatient center (*n* = 79)	*P*-value
Any complication, *n* (%)	97 (31.8%)	27 (34.2%)	0.79
Infection, *n* (%)	26 (8.5%)	4 (5.1%)	0.43
Would undergo TKA again, *n* (%)	235 (77.0%)	65 (82.3%)	0.40

Subgroup analyses: Sex and age

Subgroup analyses were performed to assess whether sex or age modified satisfaction with TKA across surgical settings. Among female patients, 73.1% of hospital TKA patients and 77.1% of outpatient TKA patients reported willingness to undergo TKA again (*P* ≈ 0.61). Among male patients, these proportions were 82.9% and 86.4%, respectively (*P* ≈ 0.58). Among younger patients (<56 years), satisfaction was similar across settings (79.2% hospital vs. 78.6% outpatient, *P* ≈ 0.92). Among older patients (≥56 years), outpatient TKA was associated with a higher proportion of patients willing to undergo TKA again (91.3% vs. 75.4%).

Multivariable logistic regression

Multivariable logistic regression models were constructed to evaluate the association between surgical setting and each primary outcome (Table 3).

**Table 3:** Multivariable logistic regression analysis evaluating predictors of postoperative outcomes following TKA, including any complication, infection, and willingness to undergo TKA again. Results are presented as adjusted odds ratios (ORs) with 95% confidence intervals (CIs) and corresponding *P*-values. Covariates included surgical setting (outpatient vs. hospital), age (per year), sex (female vs. male), body mass index (BMI, kg/m²), diabetes status (yes vs. no), surgical technique (robotic-assisted vs. conventional), and physical therapy (PT) adherence score. OR, odds ratio; CI, confidence interval; BMI, body mass index; PT, physical therapy; TKA, total knee arthroplasty

Predictor	Any complication, OR (95% CI)	P	Infection, OR (95% CI)	P	Would undergo again, OR (95% CI)	P
Outpatient vs. hospital	0.83 (0.47-1.47)	0.52	0.41 (0.13-1.27)	0.12	1.21 (0.61-2.43)	0.58
Age (per year)	0.96 (0.94-0.97)	1.1 × 10⁻⁷	0.97 (0.95-1.00)	0.047	1.00 (0.99-1.02)	0.64
Female vs. male	0.72 (0.45-1.15)	0.17	0.26 (0.11-0.61)	0.0021	0.47 (0.27-0.82)	0.0078
Robotic vs. conventional	0.75 (0.41-1.38)	0.35	0.71 (0.23-2.17)	0.54	3.29 (1.43-7.57)	0.0052
BMI (kg/m²)	1.01 (0.99-1.04)	0.41	1.07 (1.03-1.11)	0.0012	1.01 (0.98-1.04)	0.44
PT adherence score	1.27 (0.99-1.64)	0.063	1.16 (0.75-1.80)	0.50	0.47 (0.35-0.62)	2.1×10⁻⁷
Diabetes (Yes vs. No)	1.95 (1.06-3.57)	0.030	0.65 (0.21-2.00)	0.45	0.47 (0.24-0.92)	0.027

Outpatient surgical setting was not independently associated with any complication (OR 0.83, 95% CI 0.47-1.47, *P* = 0.52), infection (OR 0.41, 95% CI 0.13-1.27, *P* = 0.12), or willingness to undergo TKA again (OR 1.21, 95% CI 0.61-2.43, *P* = 0.58). These findings reflect adjusted associations within this cohort and should not be interpreted as causal effects given baseline differences between groups and the potential for residual confounding. Older age was associated with lower odds of complications (OR 0.96 per year, 95% CI 0.94-0.97, *P* ≈ 1.1 × 10⁻⁷), while diabetes was associated with increased odds (OR 1.95, 95% CI 1.06-3.57, *P* = 0.030). In the infection model, higher BMI was independently associated with increased risk (OR 1.07 per kg/m², 95% CI 1.03-1.11, *P* = 0.0012), while female sex was associated with lower odds of infection (OR 0.26, 95% CI 0.11-0.61, *P* = 0.0021). In the satisfaction model, robotic-assisted TKA was associated with higher odds of willingness to undergo TKA again (OR 3.29, 95% CI 1.43-7.57, *P* = 0.0052), while female sex (OR 0.47, 95% CI 0.27-0.82, *P* = 0.0078) and diabetes (OR 0.47, 95% CI 0.24-0.92, *P* = 0.027) were associated with lower satisfaction. A higher physical therapy adherence score was inversely associated with satisfaction (OR 0.47, 95% CI 0.35-0.62, *P* ≈ 2.1 × 10⁻⁷).

## Discussion

Among U.S. patients with isolated TKA, outpatient surgery center and hospital-based procedures were associated with similar rates of postoperative complications, infection, and willingness to undergo the procedure again. After adjustment for age, sex, BMI, diabetes, robotic technique, and physical therapy adherence, the surgical setting was not an independent predictor of any primary outcome.

These findings align with recent analyses suggesting that outpatient TKA can be performed safely in appropriately selected patients. Studies utilizing fast-track pathways and propensity-matched designs have demonstrated no significant differences in postoperative complications, readmissions, or reoperations between outpatient and inpatient TKA cohorts [17]. Similarly, prior work has demonstrated comparable, and in some cases improved, outcomes for outpatient knee and hip arthroplasty relative to inpatient procedures [12,18-19]. In contrast, earlier meta-analytic data reported higher complication rates among outpatient TKA patients, raising concerns regarding the safety of ambulatory pathways [10,20]. Differences between these findings and the present results may reflect temporal improvements in perioperative optimization, refinement of patient selection criteria, and increasing institutional experience with outpatient protocols.

Consistent with prior literature, outpatient TKA patients in this cohort were younger (mean age 47.6 ± 13.1 vs. 55.4 ± 14.9 years, *P* < 0.00001) and more frequently male (55.7% vs. 40.3%, *P* = 0.0199) than those undergoing hospital-based procedures, reflecting real-world risk stratification practices [21]. Notably, among older patients (≥56 years), those undergoing outpatient TKA reported higher satisfaction compared with their hospital-based counterparts (91.3% vs. 75.4%), suggesting that carefully selected older individuals may derive particular benefit from outpatient pathways. This is consistent with prior literature demonstrating that increasing age is associated with higher postoperative satisfaction following TKA [22].

Given the observed baseline imbalance, particularly in age and sex, these results should be interpreted within the context of selection bias inherent to outpatient arthroplasty, where lower-risk patients are preferentially selected. While multivariable adjustment was performed, residual confounding from unmeasured variables remains likely, and causal inferences regarding the effect of the surgical setting cannot be definitively established.

Infection remains a critical concern following TKA. In adjusted analyses, higher BMI (OR 1.07 per kg/m², 95% CI 1.03-1.11, *P* = 0.0012) and sex (OR 0.26 for female vs. male, 95% CI 0.11-0.61, *P* = 0.0021) were independently associated with postoperative infection, whereas surgical setting was not (OR 0.41, 95% CI 0.13-1.27, *P* = 0.12). These findings are consistent with prior evidence linking obesity to increased risk of periprosthetic joint infection and suggest that patient-level factors may play a more substantial role in infection risk than care setting [23-24]. Diabetes, although traditionally associated with infection risk, was not an independent predictor in the adjusted model (OR 0.65, 95% CI 0.21-2.00, *P* = 0.45), suggesting that its effect may be mediated through BMI and related metabolic factors.

Satisfaction, assessed as willingness to undergo TKA again, was high overall and did not differ significantly between surgical settings after adjustment (OR 1.21, 95% CI 0.61-2.43, *P* = 0.58). Instead, robotic-assisted TKA (OR 3.29, 95% CI 1.43-7.57, *P* = 0.0052) and male sex (OR 0.47 for female vs. male, 95% CI 0.27-0.82, *P* = 0.0078) emerged as significant predictors of satisfaction. Patients undergoing robotic-assisted TKA demonstrated higher odds of reporting willingness to undergo the procedure again (OR 3.29, 95% CI 1.43-7.57), which may reflect perceived or actual improvements in alignment, soft tissue balancing, or recovery experience, as well as patient expectations associated with advanced surgical technology. Female sex was associated with lower satisfaction (OR 0.47, 95% CI 0.27-0.82, *P* = 0.0078), consistent with prior literature describing sex-based differences in postoperative pain, function, and expectations following TKA [25-26].

Although diabetes was not independently associated with infection (OR 0.65, 95% CI 0.21-2.00, *P* = 0.45), it was associated with increased odds of any postoperative complication (OR 1.95, 95% CI 1.06-3.57, *P* = 0.030) and decreased likelihood of satisfaction (OR 0.47, 95% CI 0.24-0.92, *P* = 0.027). These findings suggest that patients with diabetes may experience more complex recovery trajectories and may be less likely to perceive optimal outcomes. This underscores the importance of preoperative optimization, including glycemic control and comorbidity management, as well as careful patient counseling when considering outpatient pathways.

Limitations

Several limitations should be considered. Outcomes and surgical details were self-reported, introducing the potential for recall bias and misclassification, and responses could not be validated against medical records. The sample consisted of individuals willing to complete an online survey, which may limit generalizability. Additionally, important clinical variables, including comorbidity burden beyond diabetes, American Society of Anesthesiologists (ASA) classification, operative time, anesthesia type, and perioperative protocols, were not captured and may influence outcomes. The relatively small number of infections, along with the smaller outpatient sample size, may have limited statistical power to detect differences between groups and contributed to wider confidence intervals in the infection models.

This study did not utilize validated functional outcome instruments such as the WOMAC or the KOOS, and satisfaction was assessed using a single binary measure (*willingness to undergo TKA again*). As such, outcomes were based on non-validated, self-reported measures, limiting the granularity of assessment and restricting direct comparison with studies using standardized patient-reported outcome measures (PROMs). While this approach enabled broad participation and provided real-world insight, it may not fully capture the multidimensional nature of postoperative satisfaction, including pain relief, functional improvement, and expectation fulfillment.

There was a significant baseline imbalance between the outpatient and hospital cohorts, particularly in age and sex, reflecting selection bias inherent to real-world surgical decision-making. Healthier, younger patients are more likely to be selected for outpatient TKA, limiting the internal validity of direct comparisons. Although statistical adjustment was performed, residual confounding from unmeasured variables cannot be excluded, and causal inferences regarding the effect of the surgical setting cannot be definitively established.

Preoperative osteoarthritis severity was not assessed, limiting evaluation of the influence of baseline disease burden on postoperative functional outcomes. In addition, perioperative variables, including antibiotic regimens, intravenous medication use, anesthesia, and pain management strategies, were not captured, restricting the ability to account for differences in care pathways and their potential impact on postoperative outcomes across settings.

Finally, the survey distribution methodology and reliance on self-reported data may limit reproducibility across different populations and care environments, particularly in the absence of standardized clinical validation and institutional protocol data.

Despite these limitations, this study provides real-world insight into patient-reported outcomes following TKA across diverse practice settings. Future studies may mitigate these limitations through prospective designs, incorporation of validated PROMs, and linkage with clinical or registry-based data to validate self-reported outcomes. Additionally, the use of advanced statistical approaches, such as propensity score matching or stratification using validated risk assessment tools, along with the collection of more granular perioperative and comorbidity data, would improve control of confounding and enhance the validity of comparisons between surgical settings.

## Conclusions

Among U.S. patients undergoing isolated TKA, outpatient surgery center TKA was associated with similar observed rates of complications, infection, and patient satisfaction compared with hospital-based TKA in this cohort. After adjustment for age, sex, BMI, diabetes status, surgical technique, and physical therapy adherence, the surgical setting was not an independent predictor of complications, infection, or willingness to undergo the procedure again. However, these findings should be interpreted with caution due to baseline differences between groups and the potential for selection bias and residual confounding. Higher BMI and male sex were significant predictors of postoperative infection, while robotic-assisted TKA and male sex were associated with greater odds of willingness to repeat the procedure. Diabetes was associated with an increased risk of any complication and lower satisfaction, but was not an independent predictor of infection. Overall, these findings should be considered exploratory and hypothesis-generating, highlighting the need for prospective, adequately powered studies with standardized outcome measures to further evaluate differences between outpatient and hospital-based TKA.

## Appendices

Appendix 

**Table 4:** Survey instrument for patient-reported outcomes and clinical characteristics following TKA. Source: The survey instrument was developed by the study investigators based on established patient-reported outcome domains and clinical variables relevant to total knee arthroplasty.

Section	Question	Response Options
Arthroplasty History and Eligibility	Have you ever had any of the following joint replacement surgeries? (Select all that apply)	Reverse Total Shoulder Arthroplasty (Reverse TSA); Total Ankle Arthroplasty (TAA); Total Hip Arthroplasty (THA); Total Knee Arthroplasty (TKA); Total Shoulder Arthroplasty (TSA); Wrist Arthroplasty; None of the above
	Where was your total knee arthroplasty (TKA) performed at?	Hospital; Outpatient Surgery Center
Preoperative Symptoms	How would you rate your pain and mobility level PRIOR to undergoing TKA?	0 = Extremely good; 1 = Moderately good; 2 = Neither good nor bad; 3 = Moderately bad; 4 = Extremely bad
	How would you rate the level of joint stiffness PRIOR to undergoing TKA?	0 = Extremely good; 1 = Moderately good; 2 = Neither good nor bad; 3 = Moderately bad; 4 = Extremely bad
Postoperative Symptoms and Outcomes	How would you rate your pain and mobility level AFTER undergoing TKA?	0 = Extremely good; 1 = Moderately good; 2 = Neither good nor bad; 3 = Moderately bad; 4 = Extremely bad
	How would you rate the level of joint stiffness AFTER undergoing TKA?	0 = Extremely good; 1 = Moderately good; 2 = Neither good nor bad; 3 = Moderately bad; 4 = Extremely bad
	Did you experience any post-operative complications? (Select all that apply)	Infection; Instability; Loosening; Dislocation; Fracture; Varus/Valgus Deformation; Other; None of the above
	How would you rate the improvement in your ability to exercise since your procedure?	0 = Much better; 1 = Moderately better; 2 = About the same; 3 = Moderately worse; 4 = Much worse
	If given the choice, would you choose to have this procedure again?	Yes; No
Surgical Characteristics	Was your surgery performed using robotic-assisted technology or a conventional approach?	Robotic; Conventional; Other
	Was your surgery performed using a cruciate-sparing prosthesis or a non-cruciate-sparing prosthesis?	Cruciate-Sparing Prosthesis; Non-Cruciate-Sparing Prosthesis; Other
	What type of anesthesia was used for your procedure?	General Anesthesia; Spinal or Epidural Anesthesia; Local Anesthesia (Peripheral Nerve Block); Other
Rehabilitation and Physical Therapy	Where did you complete your rehabilitation after procedure?	At-home rehabilitation; Inpatient rehabilitation facility; Outpatient rehabilitation center; Combination of at-home + inpatient; Combination of at-home + outpatient; Combination of inpatient + outpatient; None of the above
	How would you rate your adherence to physical therapy following your procedure?	0 = Excellent; 1 = Good; 2 = Average; 3 = Poor; 4 = Terrible
	How satisfied were you with the physical therapy you received after your procedure?	0 = Extremely dissatisfied; 1 = Moderately dissatisfied; 2 = Neither satisfied nor dissatisfied; 3 = Moderately satisfied; 4 = Extremely satisfied
Demographic and Clinical Characteristics	Please enter your height in inches	Free response
	What is your weight (in pounds)?	Free response
	Do you have diabetes?	Yes; No
	What was your age at the time of the procedure?	Free response
